# Supplementary material for: Design of large-span stick-slip freely switchable hydrogels via dynamic multiscale contact synergy
Source: Nat Commun. 2022 Nov 15;13:6964. doi: 10.1038/s41467-022-34816-2 (PMC9666504; doi:10.1038/s41467-022-34816-2)
Supplement: Supplementary file 1 — Supplementary Information [file 41467_2022_34816_MOESM1_ESM.pdf]

Supplementary Information for

Design of large-span stick-slip freely switchable hydrogels via dynamic multiscale contact synergy

Zhizhi Zhang[1,2], Chenxi Qin[1,2], Haiyan Feng[1,2], Yangyang Xiang[1], Bo Yu[1], Xiaowei Pei[1], Yanfei Ma[1]* and Feng Zhou[1]*

[1]State Key Laboratory of Solid Lubrication, Lanzhou Institute of Chemical Physics, Chinese Academy of Sciences, Lanzhou 730000, China.

[2]College of Materials Science and Opto-Electronic Technology, University of Chinese Academy of Sciences, Beijing, 100049, China.

*Corresponding authors. Email:

mayanfei@licp.cas.cn (Y. M.); zhouf@licp.cas.cn (F.Z.).

Table of Contents

1. Supplementary Methods 3

1.1. Chemicals and materials 3

1.2. Formulation of DMCS-hydrogels 3

2. Supplementary Figures 4

2.1. The Characterization of N-(3,4-dihydroxyphenethyl) methacrylamide (DMA) .. 4

2.2. The Characterization of copolymer poly (DMA-PFOMA) 4

2.3. The Characterization of 4-(4,8-dihydropyren-1-yl)butyl methacrylate 5

2.4. Effect of the covalent crosslink density of DMCS-hydrogels 5

2.5. Microscopic observation of DMCS-hydrogel 7

2.6. Mechanical properties of DMCS-hydrogels at different temperatures 8

2.7. Relationship between modulus and contact 9

2.8. Relationship between roughness and contact 10

2.9. The contact evolution of DMCS-hydrogel 11

2.10. Dynamic wettability of DMCS-hydrogel 12

2.11. Adhesion performance measurement of DMCS-hydrogel 14

2.12. Shear adhesion test 16

2.13. Remote Control over Adhesion Properties of DMCA-hydrogel 16

2.14. Schematic diagram of temperature control system 18

2.15. Demonstration of the adhesion strength between DMCS-hydrogel and iron sheet 18

2.16. Switchable between sticky and slippery state 19

3. Comparison of the DMCS-hydrogel and other switchable adhesive materials 20

4. Supplementary References 22

1. Supplementary Methods

1.1 Chemicals and materials. acrylic acid (AAc, >99%, TCI), acrylamide (AAm, 99%, J&K Chemical Ltd.), N-isopropylacrylamide (NIPAAm, 99%, J&K Chemical Ltd.), N, N'-methylene diacrylamide (Bis, 99%, Sinopharm Chemical Reagent Co.Ltd., China), 2-Hydroxy-4'-(2-hydroxyethoxy)-2-methylpropiophenone (2959, 98%, Energy chemical), Methacrylic Anhydride (MA, 97%, TCI), dopamine hydrochloride (DOPA-HCl, 98%, J&K Chemical Ltd.), 2,2'-azobisisobutyronitrile(AIBN, 99%, Recrystallized, aladdin), 1-Pyrenebutanol (99%, J&K Chemical Ltd.), N,N-Dimethylformamide (DMF, 99.8%, SuperDry, J&K Chemical Ltd.), 1H,1H-perfluorooctyl methacrylate (FOMA, contains MEHQ as inhibitor, 97%, J&K Chemical Ltd.) were purchased and used without any purification. Magnesium sulfate anhydrous ($MgSO_4$, ≥98%), Toluene (≥99.5%), Disodium tetraborate decahydrate ($Na_2B_4O_7 \cdot 10H_2O$, ≥99.5%), Ethyl acetate (EtOAc, ≥99.5%), Sodium chloride (NaCl, ≥99.5%), Sodium hydroxide (NaOH, ≥96%), 1, 2-Dichloroethane (≥99%), Tetrahydrofuran (THF, ≥99%), N-hexane (≥97%), 1,1,2-Trichlorotrifluoroethane (AR), Sodium hydrogen carbonate ($NaHCO_3$, ≥99.5%), hydrochloric acid (HCl) were purchased from China National Pharmaceutical Group Co., Ltd. Before utilization, NIPAAm was recrystallized from toluene.

1.2 Formulation of DMCS-hydrogels. In order to study the effect of each monomer on the performance of hydrogel. We adopted the control variable method to carry out the experiment. The pre-polymer liquid of DMCS-hydrogel is shown in the table below, where the weight percentages of AAc, AAm, NIPAAm, DMA, initiator and cross-linking agent are given with respect to the water solution. ω_w is the water weight.

Supplementary Table 1. The weight fraction of each component in the DMCS-hydrogel.

Sample code	AAc ω_1(wt%)	AAm ω_2(wt%)	NIPAAm ω_3 (wt%)	DMA ω_4 (wt%)	Cross-linker ω_5 (wt%)	Initiator Ω_6 (wt%)	Water ω_w (g)
1	3.50	3.33	10.66	0.186	0.0133	0.2	30
2	7.00	3.33	10.66	0.186	0.0133	0.2	30
3	10.51	3.33	10.66	0.186	0.0133	0.2	30
4	10.51	5.00	10.66	0.186	0.0133	0.2	30
5	10.51	6.77	10.66	0.186	0.0133	0.2	30
6	10.51	5.00	0.00	0.186	0.0133	0.2	30
7	10.51	5.00	5.33	0.186	0.0133	0.2	30
8	10.51	5.00	16.00	0.186	0.0133	0.2	30
9	10.51	5.00	16.00	0.00	0.0133	0.2	30
10	10.51	5.00	16.00	0.093	0.0133	0.2	30
11	10.51	5.00	16.00	0.186	0.0200	0.2	30
12	10.51	5.00	16.00	0.186	0.0266	0.2	30

2. Supplementary Figures

2.1 The Characterization of Dopamine methacrylamide (DMA)

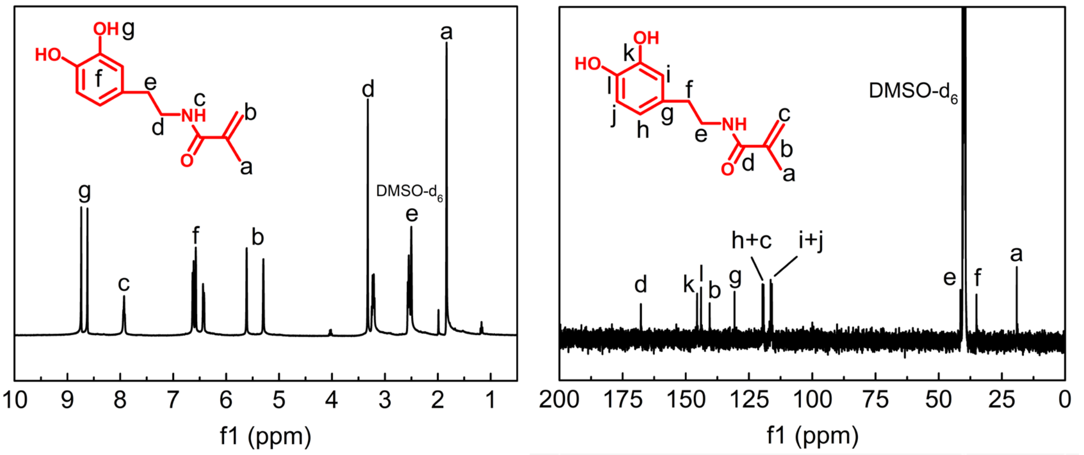

Supplementary Fig. 1 | ^{1}H-NMR spectra and ^{13}C NMR spectra of Dopamine methacrylamide (DMA) in DMSO-d_6. The chemical structure of DMA was characterized by ^{1}H NMR (left) and ^{13}C NMR (right) analysis using a Bruker AVANCE III HD NMR spectrometer. ^{1}H NMR (400 MHz, DMSO-d_6, ppm): 8.74 (s, 1H, OH), 8.62 (s, 1H, OH), 7.93 (m, 1H, NH), 7.93 (m, 1H, NH), 6.63-6.57 (m, 4H, aromatic), 5.61 (s, 1H, CH_2=), 5.29 (s, 1H, CH_2=), 3.32 (t, 2H, $NHCH_2$=), 2.50 (t, 2H, $NHCH_2CH_2$-), 1.84 (s, 3H, CH_3O-). ^{13}C NMR (400 MHz, DMSO-d_6, ppm): 167.75, 145.52, 143.96, 140.55, 130.74, 119.65, 116.43, 41.42, 35.07, 19.12. They both proved the successful synthesis of monomer DMA.

2.2 The Characterization of the copolymer poly (DMA-PFOMA)

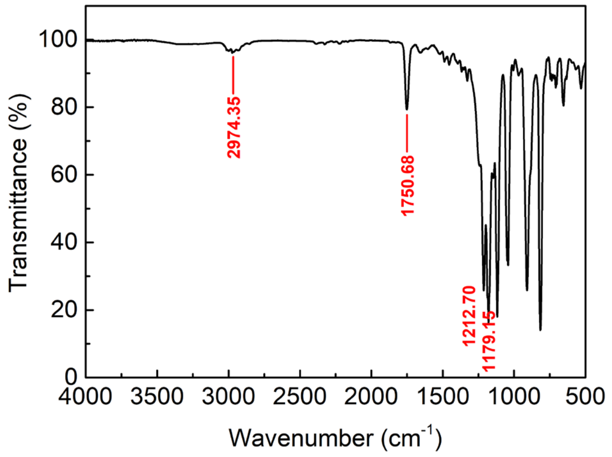

Supplementary Fig. 2 | ATR-FTIR spectra of the copolymer poly (DMA-PFOMA). The chemical characterization of poly (DMA-PFOMA) was tested by a FTIR spectrometer (Nicolet iS10, Thermo Scientific, USA). In the FTIR spectra, the characteristic peak at 2974.35 cm^{-1}, was attributed to the characteristic absorption of stretching vibration –CH_3. The peak at 1750.68 cm^{-1} corresponded to the stretching

vibration of C=O. Moreover, the peak at 1212.70 cm^{-1} and 1179.15 cm^{-1} were assigned to the stretching vibration of $-CF_3$ and C-F bond. It is proved that the polymer has been successfully synthesized.

2.3 The Characterization of 4-(1-pyrenyl) butyl methacrylate

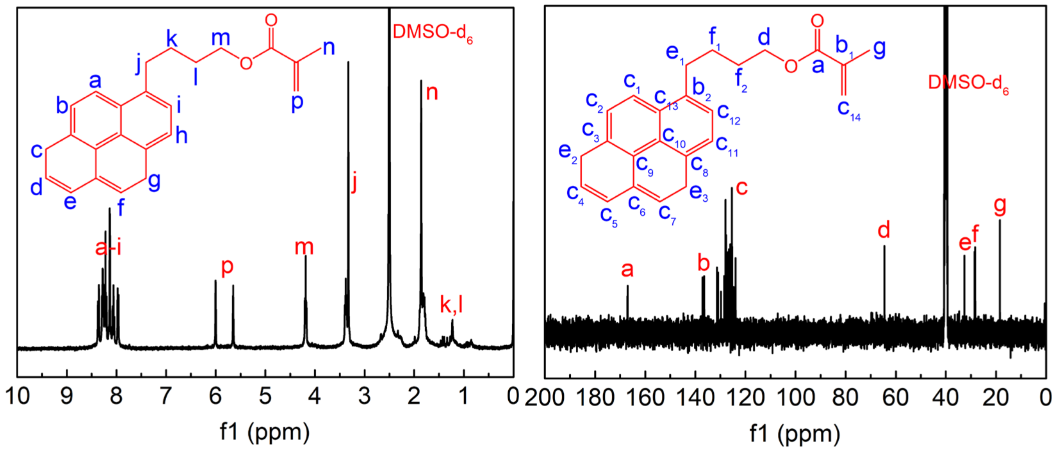

Supplementary Fig. 3 | ^{1}H-NMR spectra and ^{13}C NMR spectra of 4-(4,8-dihydropyren-1-yl)butyl methacrylate in DMSO-d$_6$. The chemical structure of it was characterized by ^{1}H NMR (left) and ^{13}C NMR (right) analysis using a Bruker AVANCE III HD NMR spectrometer. The above results proved the successful preparation of the monomer.

2.4 Effect of the covalent crosslink density of DMCS-hydrogels

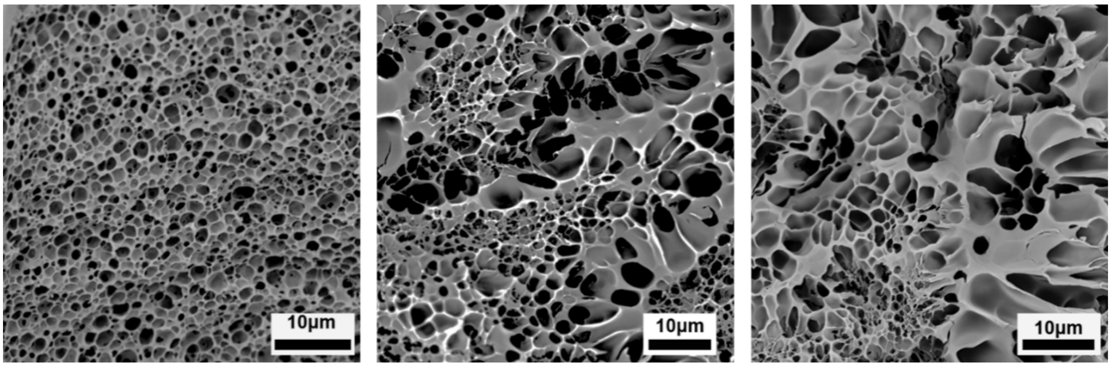

Supplementary Fig. 4 | Characterization of hydrogel microscopic morphologies. To study the effect of crosslinking degree on modulus, we prepared hybrid gels with various concentrations of the crosslinker Bis. Scanning electron microscope images are hydrogels with (left) 0.0133 wt% Bis, (middle) 0.0200 wt% Bis, (right) 0.0266 wt% Bis. As the concentration of Bis increased, the crosslink density of the network increased. Hydrogels with higher crosslinking density had smaller pores.

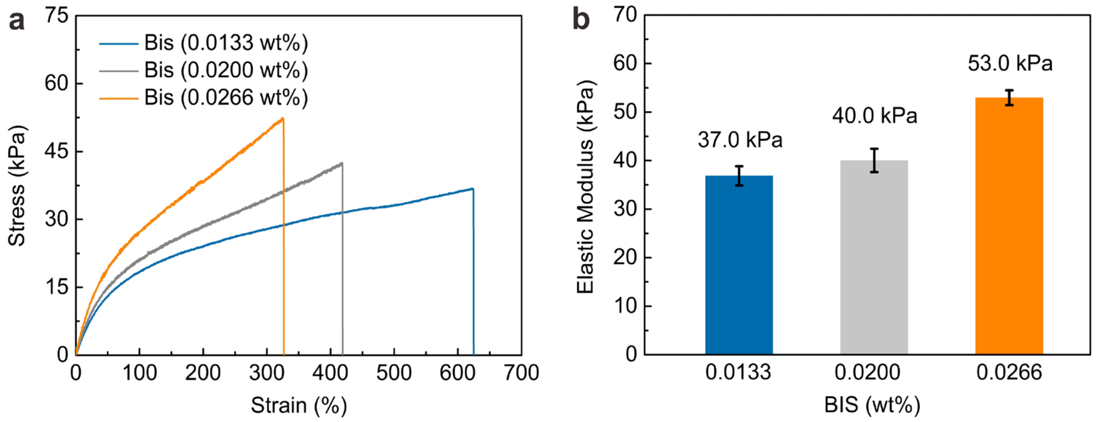

Supplementary Fig. 5 | Mechanical properties of DMCS-hydrogels with different crosslinking agent content. (a) The obtained stress versus strain curves. (b) The corresponding elastic modulus of DMCS-hydrogels. The stress-strain curves of DMCS-hydrogels were measured using an electrical universal material testing machine with a 500 N load cell (EZ-Test, Shimadzu). The concentration of Bis did greatly affect the elastic modulus of DMCS-hydrogels. The trend is as follows. As the concentration of Bis increased, the elastic modulus of the hydrogel increased. The hydrogel with lower crosslinking density (0.0133 wt% Bis) has a higher fracture strain of 625% and elastic modulus of 37 kPa. This means that the elastic modulus can be adjusted by the degree of crosslinking. Error bars represent the standard deviation from at least three replicates. Data in (b) are presented as mean values ± SD.

2.5 Microscopic observation of DMCS-hydrogel

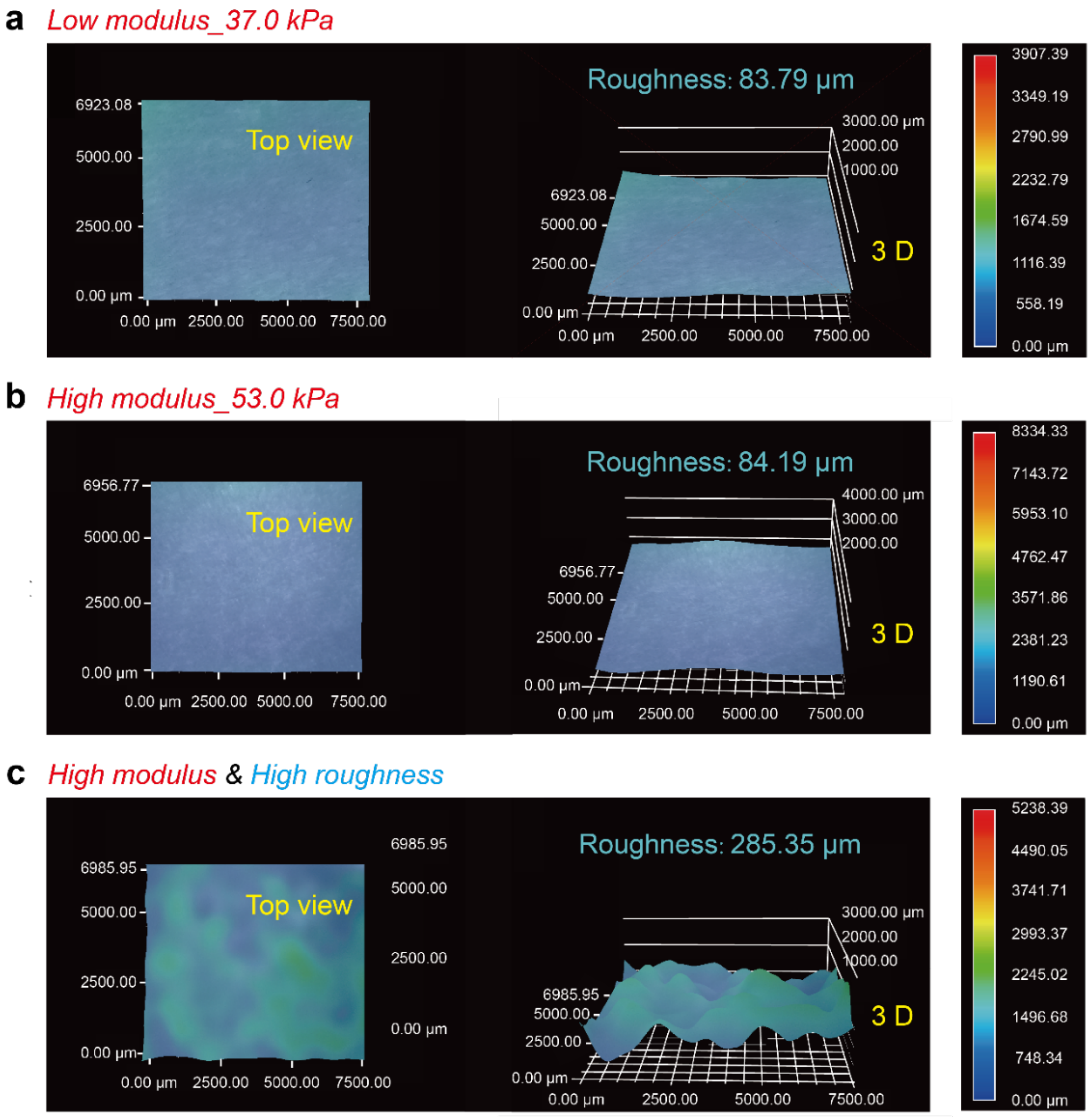

Supplementary Fig. 6 | The surface roughness parameters of DMCS-hydrogel. (a) Low modulus (b) High modulus (c) High modulus & High roughness. The area roughness parameters were measured by a 3D KEYENCE VHX-6000 digital microscope at our disposal. Microscope scanning methodology had generated the possibility of 2D and 3D analysis of the surface of the whole sample, and gave the roughness parameters of the materials. The surfaces of hydrogels were relatively smooth under the low temperature condition. However, when DMCS-hydrogel containing PNIPAAm was heated, the surface became rough because the hydrogel contracted.

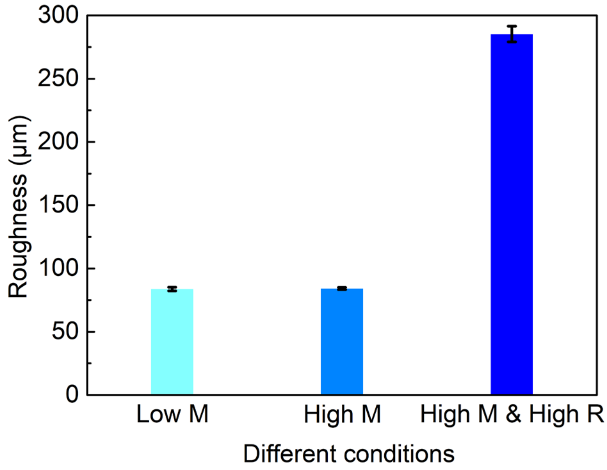

Supplementary Fig. 7 | Statistics of surface roughness parameters of hydrogels under different conditions, which is consistent with the observed surface morphology. Low M: Low Modulus; High M: High Modulus; High M & High R: High Modulus and Roughness. Error bars represent the standard deviation from at least three replicates. Data in this figure are presented as mean values ± SD.

2.6 Mechanical properties of DMCS-hydrogels at different temperatures

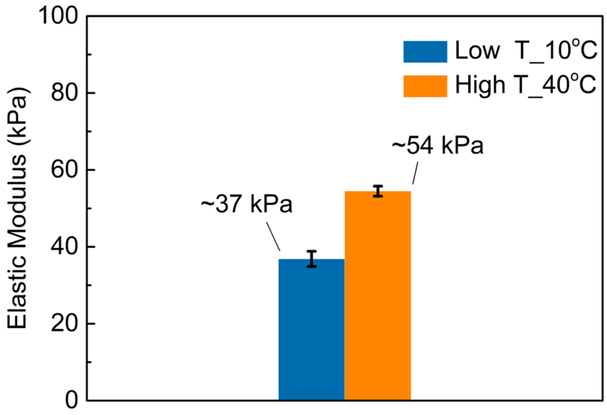

Supplementary Fig. 8 | Comparison of the mechanical properties under high and low temperature conditions. To detect the elastic modulus of hydrogels at high and low temperatures, mechanical properties were performed in hot and cold water. The water temperature was controlled at 10 °C and 40 °C, respectively. With the increase of temperature, the modulus also increased. Error bars represent the standard deviation from at least three replicates. Data in this figure are presented as mean values ± SD. T: temperature.

2.7 Relationship between modulus and contact

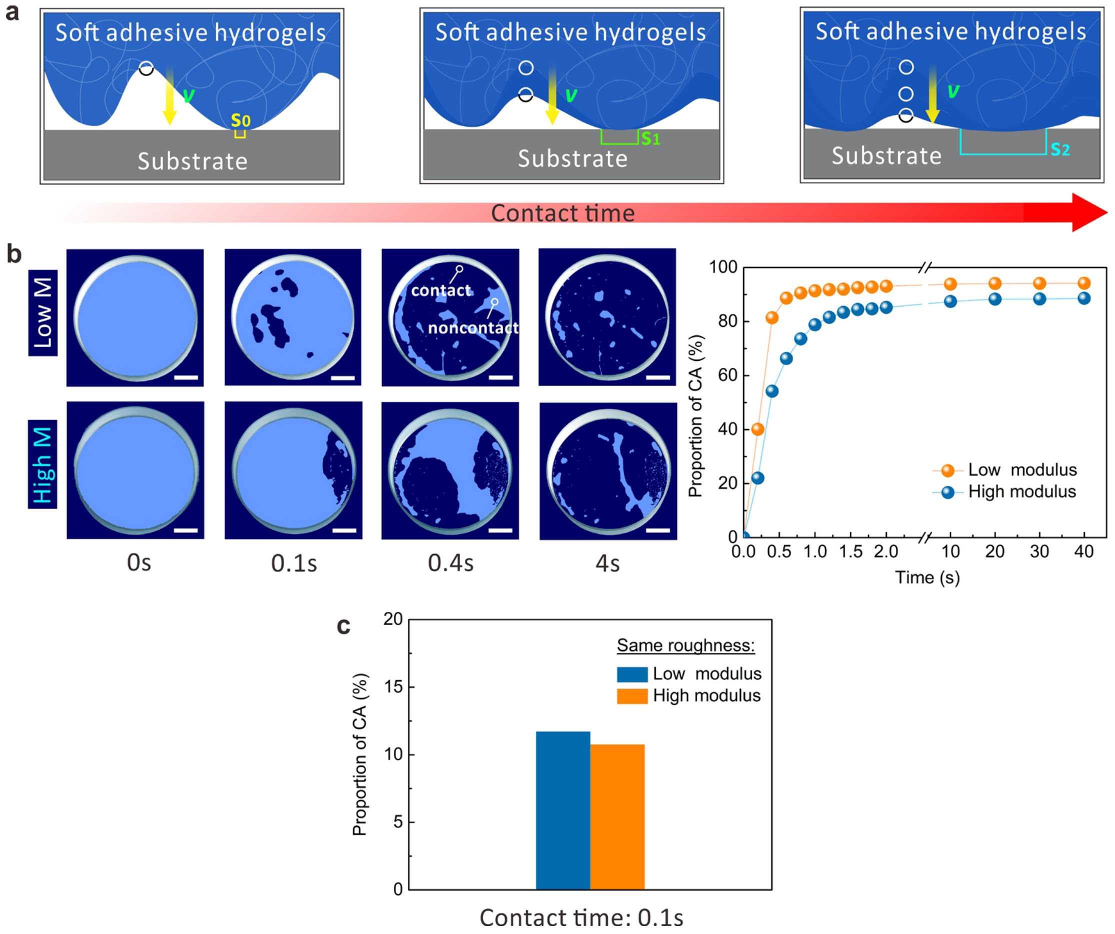

Supplementary Fig. 9 | The contact process between hydrogels (different modulus) and quartz glass. (a) Schematic diagram of contact evolution. For soft adhesive hydrogels, the actual contact is a process in which the contact area gradually increases with the extension of contact time. At the beginning, only a small number of sites can be in contact with the substrate. With the increase of contact time, there are more and more contact sites, and the contact area increases gradually. In this process, the influence of modulus on contact process cannot be ignored and is very important. Supplementary figures demonstrate the contact process between hydrogel (different modulus) and quartz glass, showing the contact conditions (b) at 0s, 0.1s, 0.4s, and 4s, and the relationship between proportion of the contacted area (CA) and time. Dark blue is the part that has been touched, and light blue is the untouched part. Here, the contact area of 0.1s is defined as the initial contact area. (c) It can be seen that the low-modulus and high-modulus hydrogels have the same initial contact area (0.1 s contact), but the low-modulus hydrogel had a faster contact rate. The above experimental results confirm that the larger the modulus is, the slower the contact speed is. v: contact velocity; $S_{0,1,2}$: contact area. Low M: Low Modulus; High M: High Modulus.

2.8 Relationship between roughness and contact

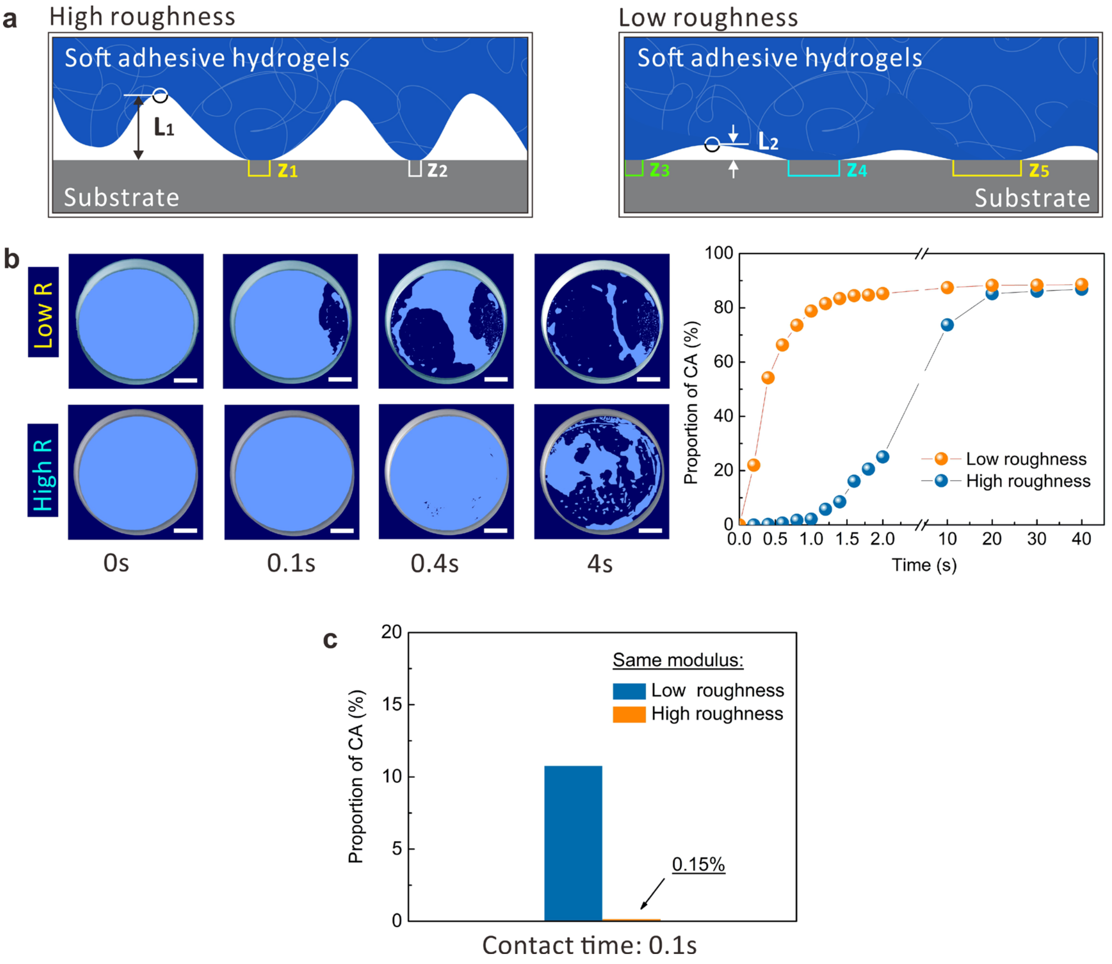

Supplementary Fig. 10 | The contact evolution between hydrogels (different roughness) and quartz glass. (a) Schematic diagram of contact evolution. For the high-roughness hydrogel, the average distance (L_1) between the hydrogel surface and the substrate is large, resulting in insufficient contact (Z_1, Z_2). However, if the roughness is low, the distance (L_2) is small, there are more contact sites at the beginning (Z_3, Z_4, Z_5), and the contact area is large. Supplementary figures demonstrate the contact process between hydrogel (different roughness) and quartz glass, showing the contact conditions (b) at 0s, 0.1s, 0.4s, and 4s, and the relationship between proportion of the contacted area (CA) and time. Dark blue is the part that has been touched, and light blue is the untouched part. Here, the contact area of 0.1s is defined as the initial contact area. (c) At 0.1s, the sample with high roughness almost has no contact with the substrate (~0.15%). Conversely, low-roughness hydrogel has a sizable initial contact area (~10%), which is in sharp contrast to the high-roughness hydrogel., indicating that roughness determined initial contact area. $L_{1,2}$: distance between the hydrogel surface and the substrate; $Z_{1,2,3,4,5}$: initial contact area between the hydrogel surface and the substrate. Low R: Low Roughness; High R: High Roughness.

2.9 The contact evolution of DMCS-hydrogel

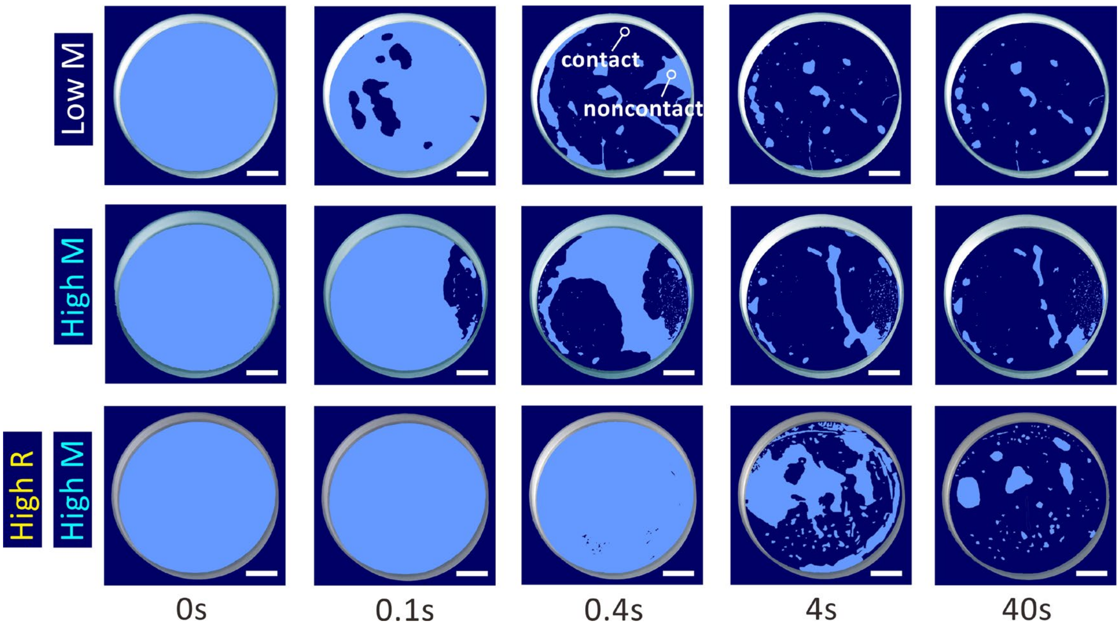

Supplementary Fig. 11 | The contact evolution between hydrogel and quartz glass under different conditions. The contact moment at 0s, 0.1, 0.4s, 4s and 40s are demonstrated. Dark blue is the part that has been touched, and light blue is the untouched part. Different conditions can be coupled and superimposed, including low modulus (Low M), high modulus (High M), high modulus (High M) & high roughness (High R).

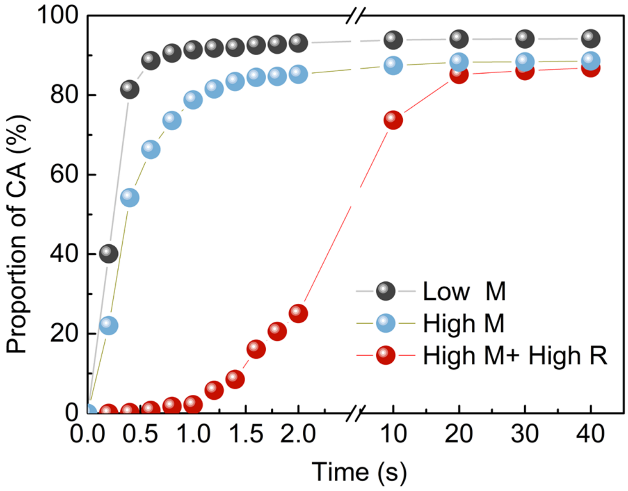

Supplementary Fig. 12 | The proportion of effective contact area with time under different conditions. This figure shows the difference intuitively. Low M: Low Modulus; High M: High Modulus; High M & High R: High Modulus and Roughness.

2.10 Dynamic wettability of DMCS-hydrogel

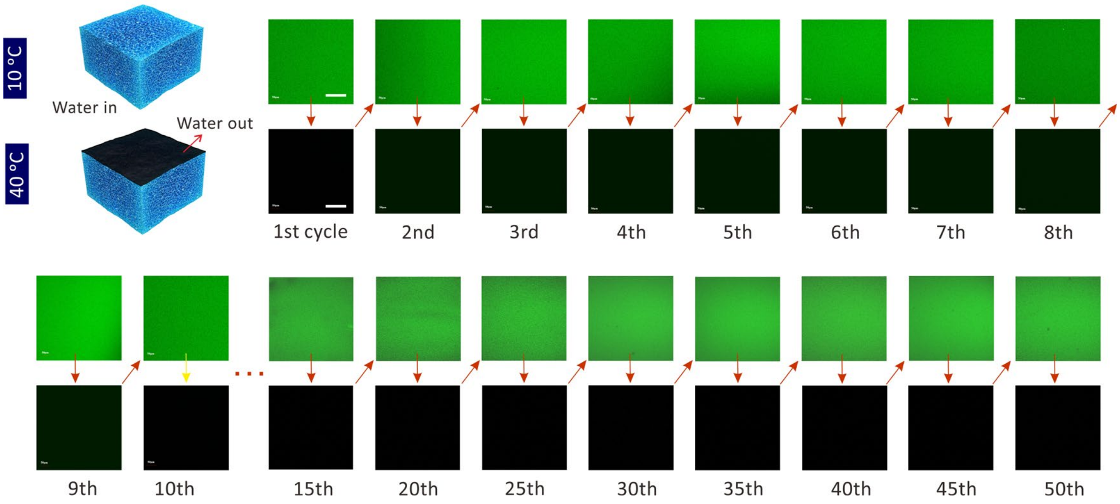

Supplementary Fig. 13 | Confocal laser scanning microscope images under low and high temperature conditions. At low temperature, the hydrogel showed strong fluorescence, but at high temperature, the fluorescence disappeared. Scale bar 100μm. The reversible cycling performance of DMCS-hydrogel does not weaken after 50 cycles. Among them, in order to prevent the hydrogel from losing water, the hydrogel was placed in water for 5 s to restore each 10 cycles, and then kept at 10 °C temperature and 60% humidity for 6 h.

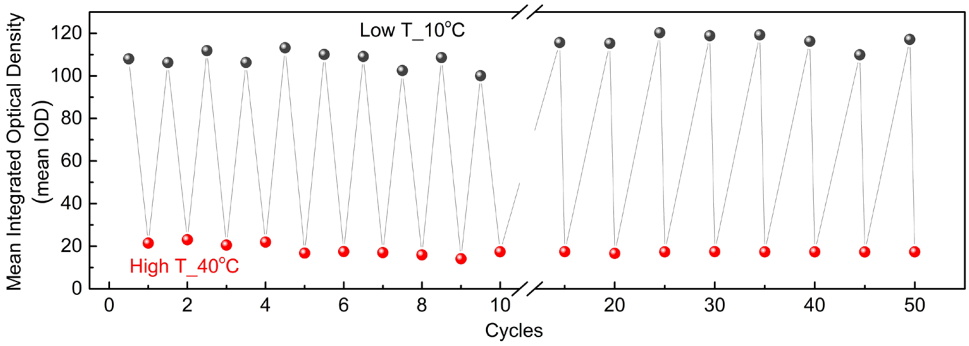

Supplementary Fig. 14 | The Mean Integrated Optical Density of hydrogels at high and low temperature conditions. The cold and hot cycles were 50 times.

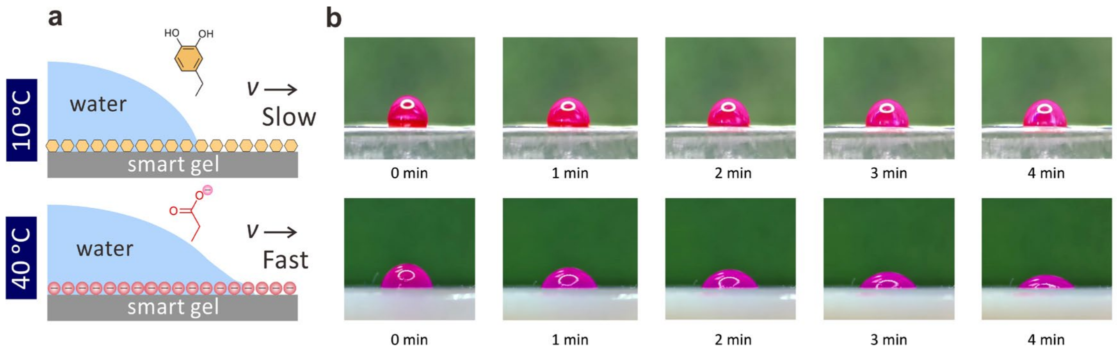

Supplementary Fig. 15 | Dynamic wetting process under high and low temperature conditions. (a) Schematic diagram of the contact angle on the high- and low-temperature conditions. (b) Change of contact angle within 4 min at high and low temperature. Water droplets (dyed by rhodamine) were dripped on the high and low-temperature hydrogels to test their dynamic wettability. v: diffusion velocity.

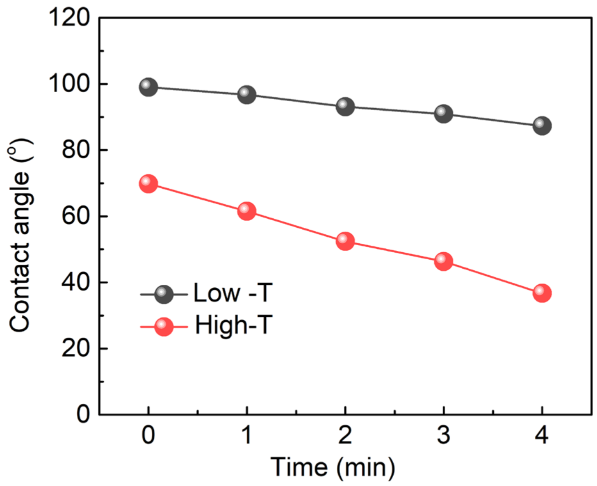

Supplementary Fig. 16 | Relationship between contact angle and time. The contact angle is smaller at high temperature than at high temperature. Water droplet spreads faster on the high-temperature DMCS-hydrogel than on the low-temperature.

2.11 Adhesion performance measurement of DMCS-hydrogel

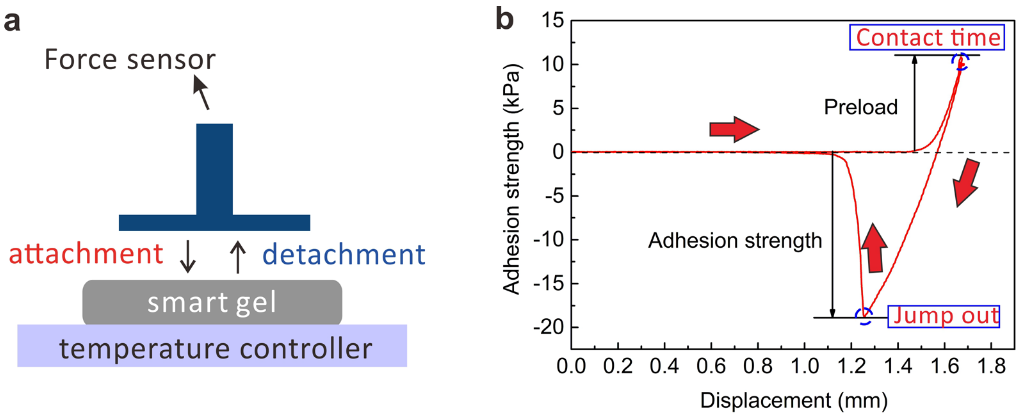

Supplementary Fig. 17 | Measure the adhesion properties of DMCS-hydrogel in face-to-face contact mode. (a) A schematic experimental setup for the adhesion measurement. (b) Displacement-adhesion strength curve. When the probe touched the hydrogel, it would apply a preload force and stop growing when it reached 1N load. After 10s, the probe would be lifted, and the absolute value of minus value was the adhesion strength.

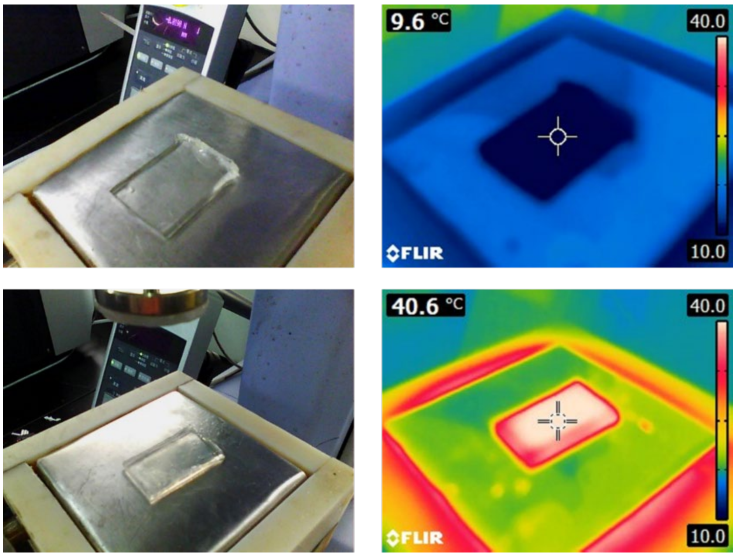

Supplementary Fig. 18 | Physical images and the corresponding infrared thermal imaging photos when cooling and heating the hydrogel. The surface of DMCS-hydrogel was cooled or heated to 10 °C and 40 °C respectively. In order to accurately control the temperature, the circulating water was used to continuously heat or cool the temperature controller, so that the adhesion strength at different temperatures can be tested.

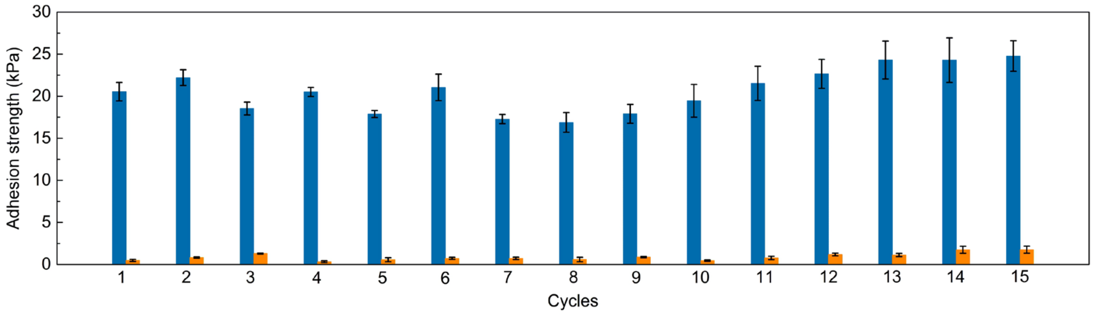

Supplementary Fig. 19 | The switchable adhesion cycles of DMCS-hydrogel at high (60 ºC) and low (10 ºC) temperature for 15 cycles. No water was added during the experiment. Error bars represent the standard deviation from at least three replicates. Data in this figure are presented as mean values ± SD.

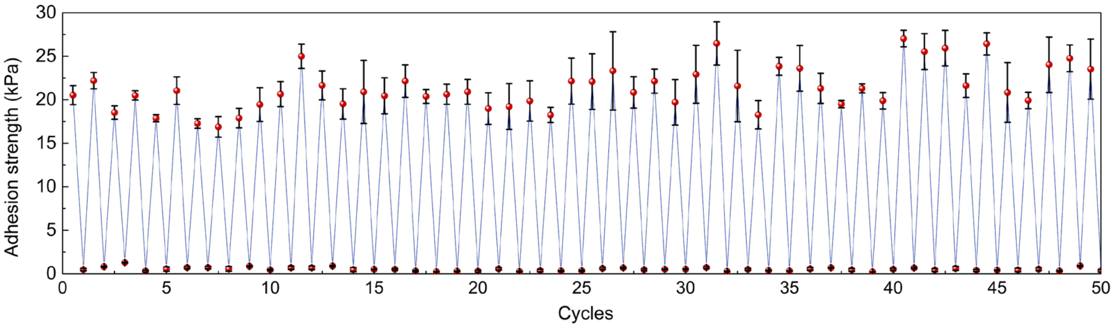

Supplementary Fig. 20 | The switchable adhesion cycles of DMCS-hydrogel at high (60 ºC) and low (10 ºC) temperature for 50 cycles (in water for 5 s, at 10 ºC temperature and 60% humidity for 6 h, every 10 cycles). Error bars represent the standard deviation from at least three replicates. Data in this figure are presented as mean values ± SD.

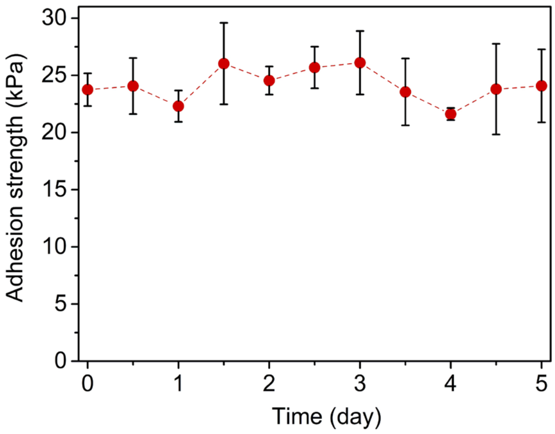

Supplementary Fig. 21 | Long-term adhesion stability of DMSC-hydrogels (10 ºC temperature and 60 % humidity). Error bars represent the standard deviation from at least three replicates. Data in this figure are presented as mean values ± SD.

2.12 Shear adhesion test

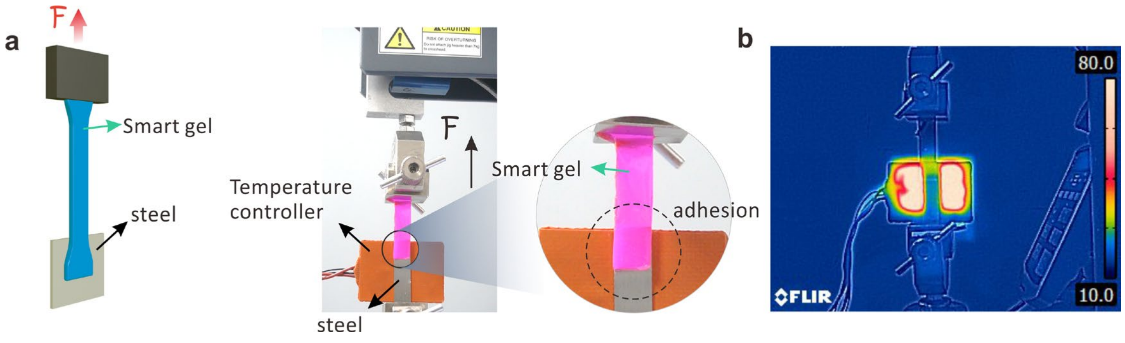

Supplementary Fig. 22 | Shear adhesion tests. (a) A shear adhesion test diagram. (b) Iron sheet and hydrogel were heated by silicone rubber heating sheet. The hydrogel was cut into strips and adhered to iron sheets, and the adhesive area is about 1cm^2. The crosshead velocity was maintained at 100 mm/min. At low temperature, the sample was fully stretched, and DMCS-hydrogel was still firmly attached to the iron sheet. However, after heating the iron sheet with silicone rubber sheet, the adhesion force became very weak. The sample slips off the surface of the iron sheet with little stretch. F: force.

2.13 Remote Control over Adhesion Properties of DMCS-hydrogel

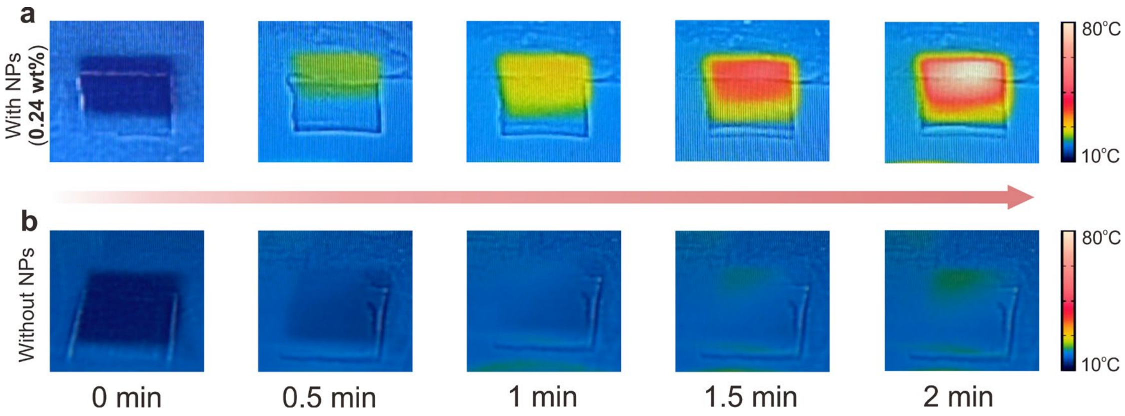

Supplementary Fig. 23 | The photothermal effect of the DMCS-hydrogels (with and without Fe_3O_4). The DMCS-hydrogels with 0.24 wt% nanoparticles (a) were gradually heated based on photothermal effect. The hydrogels without NPs were used as control samples (b).

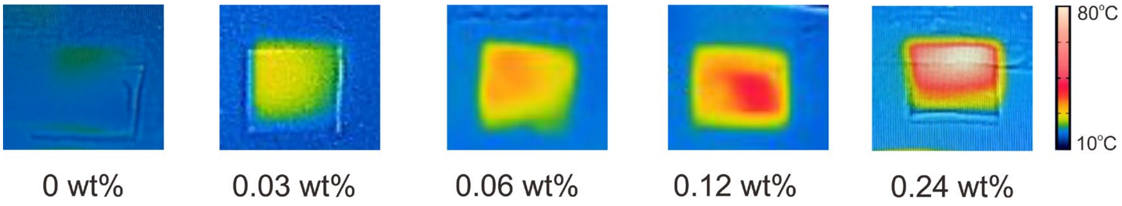

Supplementary Fig. 24 | The relationship between the temperature of hydrogels and nanoparticle content under 2 min irradiation. The temperature increased faster with the increase of the nanoparticles content. In contrast, the surface temperature of the hydrogels without nanoparticles showed hardly any increase under light irradiation.

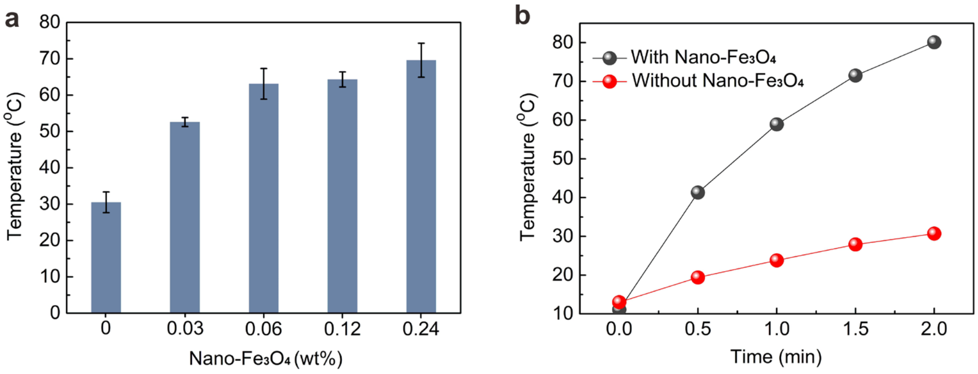

Supplementary Fig. 25 | Relationship between temperature and nanoparticle content after 2 min light irradiation. (a) Temperature after irradiation for 2min. (b) The relationship between the temperature of the DMCS-hydrogels with 0.24 wt% NPs and the irradiation time. The hydrogels without NPs were used as control samples. The temperature of the nanoparticles-containing hydrogels increases rapidly during near-infrared light for 2 min irradiation. Error bars represent the standard deviation from at least three replicates. Data in (a) are presented as mean values ± SD.

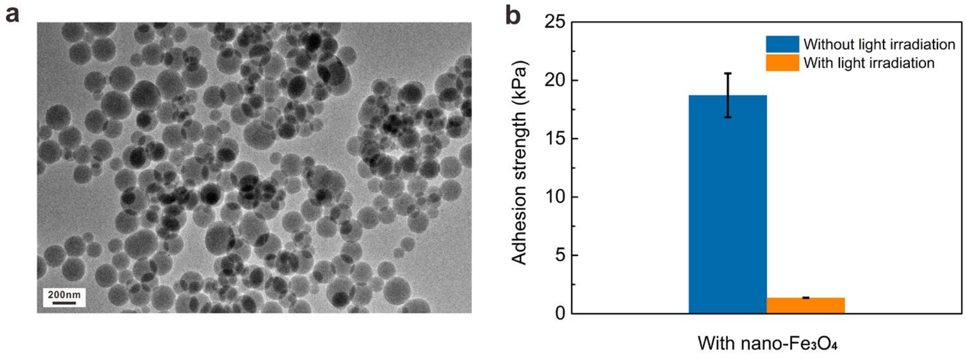

Supplementary Fig. 26 | Adhesion properties of DMCS-hydrogels (with nano-Fe_3O_4) under light irradiation and without light irradiation. (a) TEM image shows the morphology of Fe_3O_4 nanoparticles. (b) The adhesion properties of hydrogel with nano-Fe_3O_4 can be reversibly switched with and without light irradiation. Error bars represent the standard deviation from at least three replicates. Data in (b) are presented as mean values ± SD.

2.14 Schematic diagram of temperature control system

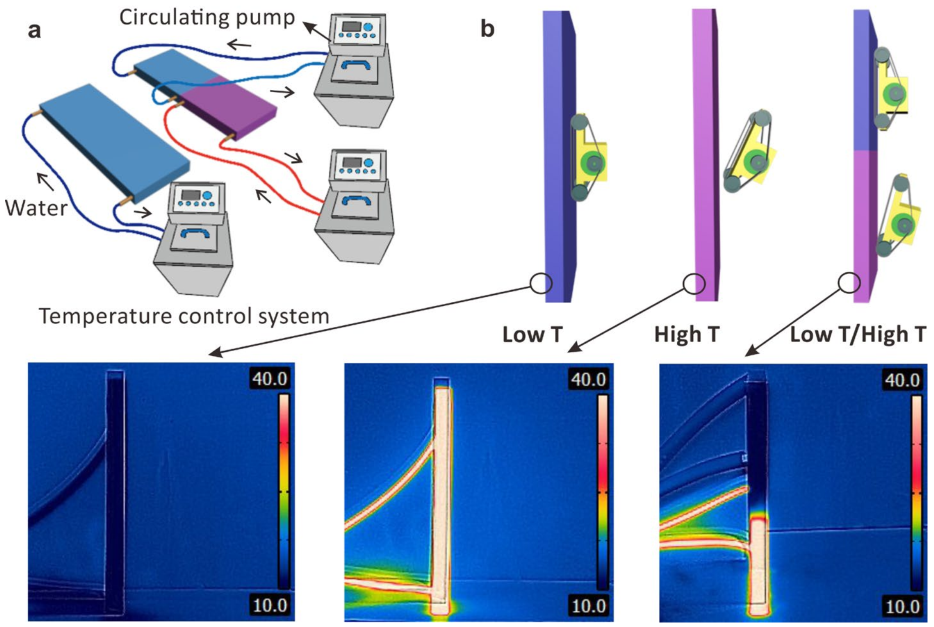

Supplementary Fig. 27 | The control of the temperature during climbing. (a) Schematic diagram of temperature control system. (b) Related infrared thermal imaging photos. The climbing robot can crawl on the nearly vertical iron plate relying on adhesion. The temperature of the metal plate can be regulated. The diagrammatic sketch shows the method for controlling the temperature of the iron plate by circulating water. T: temperature.

2.15 Demonstration of the adhesion strength between DMCS-hydrogel and iron sheet

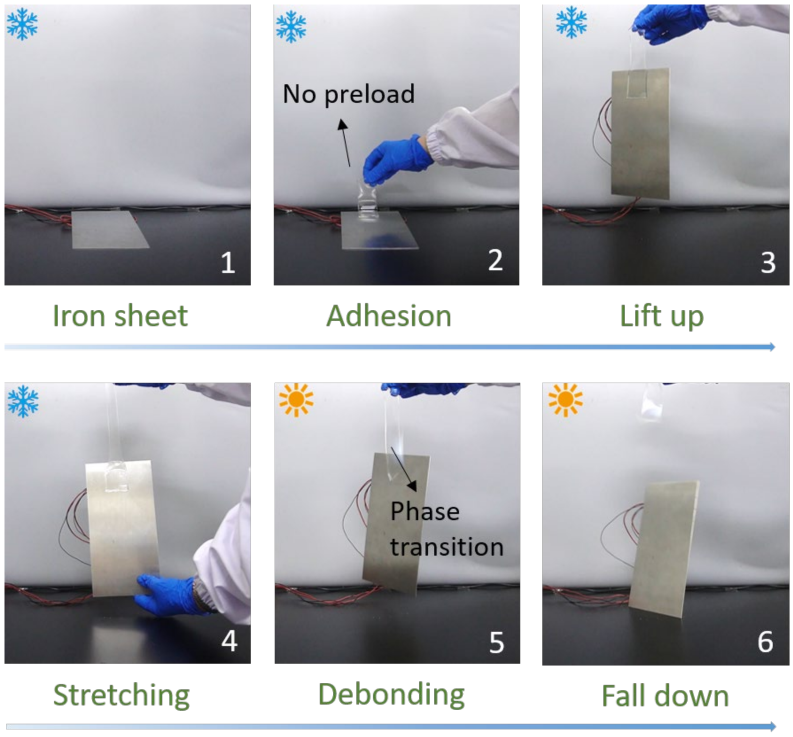

Supplementary Fig. 28 | Adhesion of DMCS-hydrogel to iron sheet. The photos show the process of lifting the iron sheet at low temperature by DMCS hydrogel and the process of drop due to insufficient adhesion at high temperature. At low temperature, hydrogel can adhere to the iron sheet after being stretched. When heating the iron piece with the heating piece, the hydrogel underwent a phase change and the adhesion gradually decreased, so the iron piece fell straight down.

2.16 Switchable between sticky and slippery state

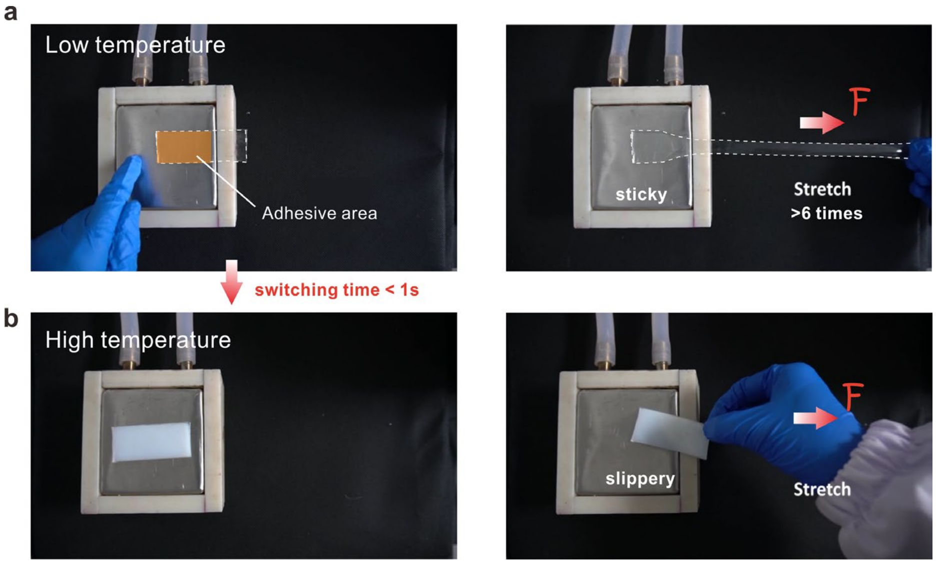

Supplementary Fig. 29 | Switching between adhesion and lubrication at high and low temperatures. (a) At low temperature, DMCS-hydrogel can be stretched to a very long length without de-bonding, which indicates that its adhesion is very strong. (b) However, at high temperature, the strategy of dynamic multiscale contact results in the hydrogel sliding on its surface. Once the low-temperature gel came into contact with the high-temperature iron sheet, the transition occurred in less than 1s. F: force.

3. Comparison of DMCS-hydrogel and other switchable adhesive materials

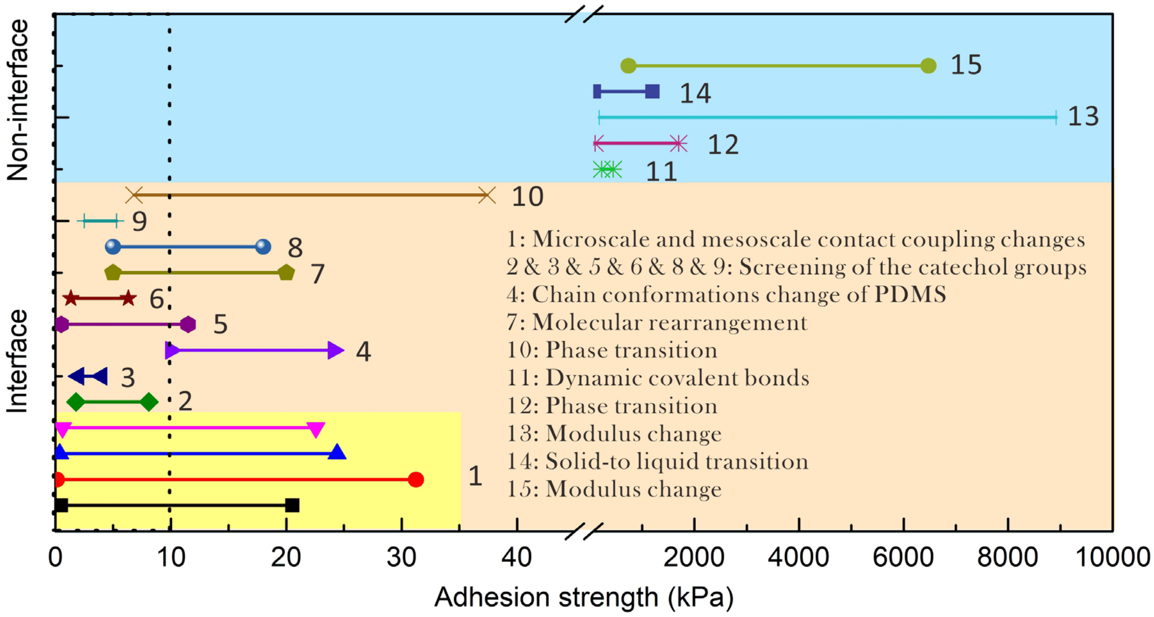

Supplementary Fig. 30 | Literature research on the switchable adhesive materials. In order to prove the superiority of the sample we designed and prepared, DMCS-hydrogel (yellow area) was compared with a verity of other switchable adhesive materials. This figure demonstrates the high and low adhesion strength of them, which are divided into interface adhesive materials (orange area) and non-interface adhesive materials (blue area).

Supplementary Table 2. Comparison of self-prepared hydrogel materials with other switchable adhesive materials.

Please note that the "--" symbol means "not mentioned in the article". Responding location: Non-interface/cohesion (**A**), Interface (mesoscale, **B1**; microscale, **B2**)

Adhesives	Materials	High adhesion	Low adhesion	Gap	Ratio	External stimulus	**Location**	Mechanism	Versatility
This work	hydrogel	31.25kPa	0.13kPa	31.12kPa	240.38	temperature	**B1, B2**	Microscale and mesoscale contact coupling changes	Wood, Ti, Fe, Al, Glass, Si, PET
Adv. Mater. 2018[1]	Polymer	8.1kPa	1.8kPa	6.3kPa	4.5	temperature	**B2**	Screening of the catechol groups	Al, Si, PP, PS, PET, PTFE, PI, Glass
Nat. Commun. 2017[2]	Polymer	4.0kPa	2kPa	2.0kPa	2.0	temperature	**B2**	Screening of the catechol groups	Al, Si, Ti, PTFE, Glass

ACS Appl. Mater. Interfaces 2019[3]	PDMS	24.1kPa	10kPa	14.1kPa	2.41	light	**B2**	Chain conformations change of PDMS	Glass
Sci. Robot. 2021[4]	Hydrogel	11.5kPa	0.5kPa	11kPa	23	electric	**B2**	Screening of the catechol groups	Hydrophilic/ Hydrophobic Glass, Ceramic, Copper, PMMA, PTFE
Langmuir. 2018[5]	Hydrogel	6.31kPa	1.343kPa	4.967kPa	4.69	pH	**B2**	Screening of the catechol groups	quartz
Nat. Commun. 2021[6]	Hydrogel	20kPa	5kPa	15kPa	4	electricity	**B2**	Molecular rearrangement	(bovine) aorta, lung, cartilage, cornea
Soft Matter. 2019[7]	Hydrogel	18kPa	5kPa	13kPa	3.6	pH	**B2**	Screening of the catechol groups	SiO_2
J. Am. Chem. Soc. 2020[8]	Polymer	5.3kPa	2.5kPa	2.8kPa	2.12	electricity	**B2**	Screening of the catechol groups	Ti
Proc Natl Acad Sci. 2020[9]	Hydrogel	400J/m^2	50 J/m^2	350J/m^2	8	solvent	**B2**	Reversible covalent crosslinking	Biological tissue
Chem. Mater. 2016[10]	Polymer	2000mJ/m^2	180mJ/m^2	1820mJ/m^2	11.11	pH	**B2**	Screening of the catechol groups	Borosilicate glass
Adv. Mater. 2019[11]	Hydrogel	200J/m^2	10J/m^2	190J/m^2	20	UV	**B2**	Phase transition	elastomers, inorganic solid, Porcine, liver, skin
Small. 2021[12]	Hydrogel	37.4kPa	6.8kPa	30.6kPa	5.5	temperature	**B1**	Phase transition	PE, Al, Steel, PTFE, Glass, PP, Rubber, porcine skin
Mater. Horiz. 2020[13]	Polymer	447.3kPa	225kPa	222.3kPa	1.988	photo- and thermo-responsive	**A**	Dynamic covalent bonds	Quartz, glass, Al, PMMA, silicone, SiC, PTFE, steel, rubber
ACS Appl. Mater. Interfaces. 2018[14]	Polymer	1.7MPa	0.1MPa	16.9MPa	17	Light	**A**	Phase transition	--
Proc Natl Acad Sci. 2019[15]	Hydrogel	8920kPa	180kPa	8740kPa	49.56	Solution	**A**	Modulus change	Glass

ACS Macro Lett. 2019[16]	Polymer	1.2MPa	0.08MPa	1.12MPa	15	Light	**A**	Solid-to-liquid transition	--
J. Am. Chem. Soc. 2019[17]	Polymer	1.34MPa	--	--	--	Green-Light	**A**	Phase transition	aluminum alloy and glass
Matter. 2021[18]	Polymer	35N/m	3N/m	32N/m	11.67	temperature	**A**	Phase transition	Al, Glass, PDMS, PU, PET, PI, skin
Chem. Eng. J. 2021[19]	Polymer	6.48Mpa	0.74Mpa	5.74Mpa	8.76	temperature	**A**	Modulus change	PTFE, PET, steel, iron, glass

4. Supplementary References

1. Ma, Y. et al. Remote Control over Underwater Dynamic Attachment/Detachment and Locomotion. *Adv. Mater.* **30**, e1801595 (2018).

2. Zhao, Y. et al. Bio-inspired reversible underwater adhesive. *Nat. Commun.* **8**, 1-8 (2017).

3. Wang, X. et al. Reversible Adhesion via Light-Regulated Conformations of Rubber Chains. *ACS Appl. Mater. Interfaces* **11**, 46337-46343 (2019).

4. Huang, J. et al. Electrically programmable adhesive hydrogels for climbing robots. *Sci. Robot.* **6**, eabe1858 (2021).

5. Narkar, A. R. & Lee, B. P. Incorporation of Anionic Monomer to Tune the Reversible Catechol-Boronate Complex for pH-Responsive, Reversible Adhesion. *Langmuir* **34**, 9410-9417 (2018).

6. Borden, L. K., Gargava, A. & Raghavan, S. R. Reversible electroadhesion of hydrogels to animal tissues for suture-less repair of cuts or tears. *Nat. Commun.* **12**, 1-10 (2021).

7. Narkar, A. R., Kendrick, C., Bellur, K., Leftwich, T., Zhang, Z. & Lee, B. P. Rapidly responsive smart adhesive-coated micropillars utilizing catechol-boronate complexation chemistry. *Soft Matter* **15**, 5474-5482 (2019).

8. Akram Bhuiyan, M. S., Roland, J. D., Liu, B., Reaume, M., Zhang, Z., Kelley, J. D. & Lee, B. P. In situ deactivation of catechol-containing adhesive using electrochemistry. *J. Am. Chem. Soc.* **142**, 4631-4638 (2020).

9. Chen, X., Yuk, H., Wu, J., Nabzdyk, C. S. & Zhao, X. Instant tough bioadhesive with triggerable benign detachment. *Proc. Natl. Acad. Sci. USA* **117**, 15497-15503 (2020).

10. Narkar, A. R., Barker, B., Clisch, M., Jiang, J. & Lee, B. P. pH Responsive and Oxidation Resistant Wet Adhesive based on Reversible Catechol-Boronate Complexation. *Chem. Mater.* **28**, 5432-5439 (2016).

11. Gao, Y., Wu, K. & Suo, Z. Photodetachable Adhesion. *Adv. Mater.* **31**, e1806948 (2019).

12. Shi, X. & Wu P. A Smart Patch with On-Demand Detachable Adhesion for Bioelectronics. *Small* **17**, e2101220 (2021).

13. Wang, Z., Guo, L., Xiao, H., Cong, H. & Wang, S. A reversible underwater glue based on photo- and thermo-responsive dynamic covalent bonds. *Mater. Horiz.* **7**, 282-288 (2020).

14. Ito, S., Akiyama, H., Sekizawa, R., Mori, M., Yoshida, M. & Kihara, H. Light-Induced Reworkable Adhesives Based on ABA-type Triblock Copolymers with Azopolymer Termini. *ACS Appl. Mater. Interfaces* **10**, 32649-32658 (2018).

15. Cho, H. et al. Intrinsically reversible superglues via shape adaptation inspired by snail epiphragm. *Proc. Natl. Acad. Sci. USA* **116**, 13774-13779 (2019).

16. Zhou, Y. et al. Light-Switchable Polymer Adhesive Based on Photoinduced Reversible Solid-to-Liquid Transitions. *ACS Macro Lett.* **8**, 968-972 (2019).

17. Wu, Z. et al. Green-Light-Triggered Phase Transition of Azobenzene Derivatives toward Reversible Adhesives. *J. Am. Chem. Soc.* **141**, 7385-7390 (2019).

18. Gao, M. et al. Skin temperature-triggered, debonding-on-demand sticker for a self-powered mechanosensitive communication system. *Matter* **4**, 1962-1974 (2021).

19. Zhang, M-H., Li, C-H. & Zuo, J-L. A variable stiffness adhesive enabled by joule heating effect. *Chem. Eng. J.* **433**, 133840 (2022).
