# Supplementary material for: Design of large-span stick-slip freely switchable hydrogels via dynamic multiscale contact synergy
Source: Nat Commun. 2022 Nov 15;13:6964. doi: 10.1038/s41467-022-34816-2 (PMC9666504; doi:10.1038/s41467-022-34816-2)
Supplement: Supplementary file 3 — Description of Additional Supplementary Files [file 41467_2022_34816_MOESM3_ESM.pdf]

Description of Additional Supplementary Files

File name: Supplementary Movie 1

Description: The contact evolution of DMCS-hydrogel. We observed the contact evolution of hydrogel and quartz glass within 40s. At low temperature, quartz glass can be rapidly contacted with hydrogel. But it took a long time at high temperature, about 100 times longer than that at low temperature.

File name: Supplementary Movie 2

Description: The dynamic wetting behaviour on DMCS-hydrogel surface. Fluorescent droplets were dripped on the surface of the hydrogel. An OLYMPUS optical microscope recorded and captured movies. When heating the DMCS-hydrogel, the reorientation of the hydrophobic and hydrophilic groups leads to the dynamic wetting speed of fluorescent droplets faster than that at low temperature.

File name: Supplementary Movie 3

Description: Switch between sticky and slippery state. DMCS-hydrogel had strong adhesion at low temperature and can be stretched to a very long distance. However, at high temperature, the hydrogel rapidly switched from high adhesion to a lubricating state. As long as a small force was applied to the hydrogel at high temperature, the hydrogel can slide anywhere on the platform, so the hydrogel can easily detach from the substrate.

File name: Supplementary Movie 4

Description: Shear adhesion test. In addition to using the face-to-face contact mode to test the adhesion strength, we also tested the shear adhesion strength. The adhesion area was about 1cm^2, and the crosshead velocity was maintained at 100 mm/min. The hydrogel could be stretched to about 6.5 times the original length without debonding at low temperature. However, at high temperature, the adhesion between hydrogel and iron sheet was very weak. The hydrogel rapidly stripped from the iron sheet when it was stretched.

File name: Supplementary Movie 5

Description: The fast response of smart mobile device. Two thermostatic controllers were used to control the temperature of metal plate, which was divided into two temperature regions. The movie exhibits the robot climbing on a half-cold and half-hot substrate. The substrate was nearly vertical. At low temperature, the adhesion of DMCS-hydrogel was sufficient to support the movement of climbing robot, but it did not work in the high temperature region.
